# Experimental evolution of a sexually selected display in yeast

**DOI:** 10.1098/rspb.2008.1146

**Published:** 2008-10-07

**Authors:** David W. Rogers, Duncan Greig

**Affiliations:** 1 Division of Cell and Molecular Biology, Imperial College of Science, Technology and MedicineSir Alexander Fleming Building, Imperial College Road, London SW7 2AZ, UK; 2 Genetics, Evolution and Environment, University College LondonWolfson House, 4 Stephenson Way, London NW1 2HE, UK

**Keywords:** sexual selection, experimental evolution, *Saccharomyces cerevisiae*, mate choice, sexual display

## Abstract

The fundamental principle underlying sexual selection theory is that an allele conferring an advantage in the competition for mates will spread through a population. Remarkably, this has never been demonstrated empirically. We have developed an experimental system using yeast for testing genetic models of sexual selection. Yeast signal to potential partners by producing an attractive pheromone; stronger signallers are preferred as mates. We tested the effect of high and low levels of sexual selection on the evolution of a gene determining the strength of this signal. Under high sexual selection, an allele encoding a stronger signal was able to invade a population of weak signallers, and we observed a corresponding increase in the amount of pheromone produced. By contrast, the strong signalling allele failed to invade under low sexual selection. Our results demonstrate, for the first time, the spread of a sexually selected allele through a population, confirming the central assumption of sexual selection theory. Our yeast system is a powerful tool for investigating the genetics of sexual selection.

## 1. Introduction

Sexual selection arises from differences in reproductive success caused by competition over mates (Andersson 1994). Any trait that influences success in these competitions will be subject to sexual selection; initially, rare mutations to sexual traits are expected to spread through a population if they increase reproductive success. This simple idea forms the basis of sexual selection theory, yet has never been directly observed.

Theoretical models, based on few-locus or quantitative genetics, have revealed a number of possible genetic mechanisms underlying the evolution of sexual traits (reviewed in Maynard Smith 1991). Although these models are based on specific assumptions and make clear predictions, they are extremely difficult to test empirically. The organisms typically used to study sexual selection, such as birds, insects and fishes, have long generation times, making accurate measures of fitness extremely difficult. Moreover, most are currently genetically intractable and the genetic architecture of their sexual traits is poorly understood. Consequently, sexual selection research is largely restricted to a speculative ‘top-down’ approach of inferring genetic causes from phenotypic patterns (Andersson & Simmons 2006). Attempting to determine how an existing sexual trait evolved is complicated by two factors: (i) multiple mechanisms probably operate simultaneously and (ii) different models often predict similar outcomes. The inability to evaluate the claims of the rapidly proliferating number of theoretical models has resulted in increasing confusion over how sexual selection actually operates (Kokko *et al.* 2006), and has been blamed for the abandonment of old untested models in favour of newer, but not necessarily better, alternatives (Pizzari & Snook 2003; Kotiaho & Puurtinen 2007). Clearly, there is a great need for a new empirical model system that allows direct observation of the evolution of a sexual trait under rigorously controlled conditions.

Progress in testing other evolutionary and ecological theories has been greatly advanced in recent years by studies using laboratory populations of micro-organisms (e.g. Lenski & Travisano 1994). In particular, the yeast *Saccharomyces cerevisiae* has become the principal model system for the study of the evolution of the eukaryotic genetic system (reviewed in Zeyl 2006). This species is ideally suited to experimental studies of evolution: it has large population sizes, short generation times (allowing the accurate measure of fitness and the ability to observe evolution in action) and is associated with a vast array of genetic and genomic technology. It also has enormous, but entirely untapped, potential for the study of sexual selection (Pagel 1993).

Mate choice in yeast is simple and well documented (Jackson & Hartwell 1990*a*,*b*). Courtship occurs between haploid cells of two different mating types. *MATa* and *MATα* cells respond to the sexual signal produced by the other mating type (α- or *a*-pheromone, respectively), resulting in conjugation between two cells of different mating types and the formation of a diploid zygote. When given the choice between two potential mating partners, the cells of both mating types polarize in the direction of the highest pheromone concentration; they choose to mate with the cell producing the strongest signal.

Several aspects of the yeast mating system have prevented the use of *S. cerevisiae* as a model organism for the study of sexual selection. Sex cells are produced in tetrads that consist of the four haploid cells from a single meiosis. Each tetrad is bound together by an envelope, the ascus, and contains two *MATa* and two *MATα* cells that usually mate with each other (self-fertilization), rather than with the cells from other tetrads. Any unmated haploid can divide by mitosis and then switch mating type, allowing it to mate with the cell it just produced. These mechanisms reduce the potential for sexual selection in two ways. First, mating between genetically related individuals should evolve to be as efficient as possible (Maynard Smith & Harper 2003), minimizing the cost, and therefore the strength, of the pheromone signals. Second, the balanced mating-type ratio arising from meiosis and the physical proximity ensured by the ascus mean that cells can find partners easily, so competition for mates is minimal. Here, we describe an experimental evolution system (figure 1) that lifts these constraints on sexual selection.

The power and potential of our system is demonstrated here by the experimental evolution of a sexual signal. We show, for the first time, the spread of a strong signalling allele driven by mate choice. One of the haploid yeast mating types, *MAT*α, was designated the signaller (typically the ‘male’ role), and the other, *MATa*, as the receiver (typically the ‘female’ role). We introduced a rare strong signalling allele, which increased α-pheromone production, into an otherwise isogenic population of weak signallers. This ancestral population was used to found 12 replicate experimental populations: six exposed to high sexual selection and six to low sexual selection. To generate high sexual selection, an excess of signaller cells was mixed with rare receiver cells to promote competition between signallers for mates. To generate low sexual selection, rare signaller cells were mixed with excess receiver cells to ensure high numbers of mating opportunities for each signaller. Both the frequency of the strong signalling allele and the strength of the α-pheromone sexual signal itself were monitored as the populations evolved over 13 experimental cycles.

## 2. Material and methods

### (a) Manipulation of signal strength

Wild-type yeasts have two genes encoding the α-pheromone—*MFα1* and *MFα2*—but the majority of pheromone is produced at the *MFα1* locus. To generate weak signallers, we replaced *MFα1* with the marker *KanMX4*, which confers resistance to the drug G418. Thus, weak signallers only produced α-pheromone from the less powerful *MFα2* gene. Strong signallers produced α-pheromone from both the *MFα2* gene and the intact *MFα1* gene (and therefore lacked G418 resistance). The amount of α-pheromone produced was monitored using the halo assay (Jackson & Hartwell 1990*a*).

### (b) Strains and media

The construct *can1::MFA1pr-HIS3-MFα1pr-LEU2*, which expresses *HIS3* only in *MATa* cells and *LEU2* only in *MAT*α cells, was used to selectively propagate one or other mating type (Tong *et al.* 2001). This construct was generously provided by Charles Boone (University of Toronto) in strain Y3598 (*MATa can1::MFA1pr-HIS3-MFα1pr-LEU2 his3Δ1 leu2Δ0 ura3Δ0 met15Δ0*), which is derived from S288c (Mortimer & Johnston 1986; Brachmann *et al.* 1998). This strain was crossed to YDG577 (*MAT*α, *ade2*), which is isogenic with Y55 (McCusker & Haber 1988), and segregants of this cross were backcrossed three more times to YDG577 to create strain YDG1067 (*MATa can1::MFA1pr-HIS3-MFα1pr-LEU2 his3Δ1 leu2Δ0 ura3Δ0*), which, unlike S288c, sporulates well. All strains used in the experimental evolution study were isogenic with YDG1067. YDG1067 was transformed with plasmid pDG150 (2 μM *HO LEU2*), which allowed it to switch mating type, producing a homozygous diploid whose tetrads were dissected to obtain a *MATα* plasmid-free isogenic segregant. PCR-mediated gene replacement and crossing were used to generate the weak signalling allele *mfα1::KanMX4* in this background producing the four isogenic ancestral strains: YDG1074 (*MAT*α *can1::MFA1pr-HIS3-MFα1pr-LEU2 his3Δ1 leu2Δ0 ura3Δ0*); YDG1075 (*MATa can1::MFA1pr-HIS3-MFα1pr-LEU2 his3Δ1 leu2Δ0 ura3Δ0*); YDG1093 (*MAT*α *mfα1::KanMX4 can1::MFA1pr-HIS3-MFα1pr-LEU2 his3Δ1 leu2Δ0 ura3Δ0*); and YDG1096 (*MATa mfα1::KanMX4 can1::MFA1pr-HIS3-MFα1pr-LEU2 his3Δ1 leu2Δ0 ura3Δ0*). The signal strength in these four strains is shown in figure 2.

To make an α-factor hypersensitive strain to use to assay signal size, PCR-mediated gene replacement was used to delete *BAR1* from strain Y06055 (EUROSCARF project, Institut für Molekulare Biowissenschaften, Frankfurt, Germany), creating strain YDG1121 (*MATa bar1::URA3 sst2::KanMX4 his3Δ1 leu2Δ0 ura3Δ0 met15Δ0*), which is isogenic with S288c.

Standard media recipes and yeast methods were used throughout (Burke *et al.* 2000). Strains were grown at 30°C on YEPD or synthetic complete medium lacking either leucine (Leu-Dropout for propagation of *MAT*α strains) or histidine (His-Dropout for propagation of *MATa* strains). Sporulation medium was 2 per cent potassium acetate supplemented with synthetic complete amino acid mix. Solid media included 2 per cent agar in 25 ml Petri dishes, and liquid media were shaken 5 ml cultures.

### (c) Experimental cycle

The experimental evolution system was carried out over 13 cycles, as described in figure 1. The two *MAT*α and the two *MATa* ancestral strains were grown to stationary phase overnight in shaken 5 ml liquid cultures of Leu-Dropout or His-Dropout, respectively. These were used to prepare six mixtures of *MAT*α cells containing 1 per cent strong (YDG1074) and 99 per cent weak signallers (YDG1093) by volume and six mixtures of *MATa* cells containing 1 per cent strong signal carriers (YDG1075) and 99 per cent weak signal carriers (YDG1096) by volume. A sample from each of these 12 mixtures was taken to determine the initial frequency of the strong signalling allele in each (see §2*d*). The *MAT*α and *MATa* mixtures were paired, and each of the six pairs were used to found a high sexual selection line (99% *MAT*α, 1% *MATa*) and a low sexual selection line (1% *MAT*α, 99% *MATa*). From each of the 12 mixtures, 50 μl was pipetted onto the surface of a YEPD plate and incubated overnight to allow mating and diploid formation. The wide ends of sterile 1 ml blue pipettor tips were used to punch out 12 plugs of agar from the 12 patches of mated cells, and each was transferred into 5 ml liquid sporulation medium and incubated for 2 days. The cultures were then heated at 55°C for 30 min to kill unmated haploids and unsporulated diploids. Fifty microlitre samples of the 12 purified spore cultures were then used to inoculate 12 tubes of 5 ml Leu-Dropout and 12 tubes of His-Dropout. These tubes were incubated for 2 days to allow selective germination and growth to stationary phase of one or other mating type. The mating types were then mixed in the appropriate proportions and allowed to mate as before. This cycle was repeated a further 12 times.

### (d) Allele frequency assay

The frequency of the strong allele was determined at each cycle by serially diluting samples from the 12 Leu-Dropout *MAT*α haploid cultures and plating them onto YEPD plates, so that they yielded approximately 100 colonies after 2 days of incubation. These colonies were replica plated to YEPD-G418 (YEPD supplemented with 300 mg l^−1^ of the drug G418, which only permits the cells with the *mfα1::KanMX4* to grow) and incubated for a further day before the colonies on each plate were counted and used to estimate the frequency of the weak and strong signalling alleles in each lineage.

### (e) Signal size assay

The production of α-pheromone was visualized by pipetting a 50 μl sample of the haploid liquid culture to be tested onto the surface of a YEPD plate and incubating for 1 day to allow it to form a patch of cells. The plate was then sprayed with an aerosol of α-factor hypersensitive strain YDG1121. After a further day's incubation, YDG1121 formed a lawn of cells on the surface of the plate, with a clear ‘halo’ of growth inhibition around the patch. The size of the halo indicated the amount of pheromone production.

### (f) Fecundity assay

Mating and sporulation efficiency of the strong and weak signallers were compared by setting up matings as for high sexual selection treatment, but with each signaller in isolation, without competition between the strains bearing the different signalling alleles. Ten mixtures containing 1 per cent *MATa* strain 1075 mixed with 99 per cent of either the *MAT*α strong (1074) or the *MAT*α weak signaller (1093) were prepared from stationary phase liquid cultures, allowed to mate on the surface of YEPD plates, sporulated and purified by heat-killing unsporulated cells, just as in the evolution experiment. The number of viable spores produced by the two strains was determined by serially diluting and plating the cells on YEPD, and counting the colonies that resulted after 3 days of incubation.

### (g) Vegetative haploid fitness assay

The asexual fitness of the strong signaller relative to the weak signaller was assayed by direct competition (Lenski *et al.* 1991). Both *MAT*α strains 1074 and 1093 were grown to stationary phase in Leu-Dropout liquid cultures and mixed in equal volumes. A sample of this mixture was taken and the initial frequency of the strong signaller was determined by serial dilution and plating as for the allele frequency assay. The mixture was then diluted 10^−6^ into 5 ml fresh Leu-Dropout medium, grown to stationary phase and the final frequency of the strong signaller was determined as before. The asexual fitness of the strong signaller relative to the weak signaller is determined from the change in frequency and is given by the ratio of Malthusian parameters (Lenski *et al.* 1991).

### (h) Mate choice assay

This assay measured the change in frequency of the weak signaller allele due to receiver preference. Thus, when assaying the evolved populations, it was necessary to ensure that only receivers that did not already carry the weak signaller allele were included. Therefore, representative cultures of the receivers that had evolved under high sexual selection were generated by isolating single colonies from the samples of signallers from the *MATa* His-Dropout stationary phase cultures following the 11th round of mating. Individual colonies were screened for resistance to G418, and 20 G418-sensitive colonies from each of the six evolved replicates were picked into six tubes containing 5 ml His-Dropout and grown to stationary phase. Six cultures of the ancestral receiver strain YDG1075 were also prepared. A 50 per cent mixture of the strong (YDG1074) and weak signallers (YDG1093) was made by mixing equal volumes of stationary phase Leu-Dropout cultures of the two strains. The initial frequency of the two signaller types in this mixture was determined by the same method as the allele frequency assay, but the assay was repeated six times to increase its accuracy. The mixture of the two signallers was then mixed with three samples of each of the 12 cultures of receivers (six ancestral and six evolved) in a 99 : 1 ratio (signallers : receivers). The 36 resulting cultures were treated as in §2*c*: they were allowed to mate and form spores, from which pure cultures of *MATa* cells were propagated in His-Dropout. The frequency of the weak signaller allele was determined by diluting and plating *MATa* cells from these cultures onto YEPD plates and replica plating the resulting colonies to YEPD-G418 plates to determine the frequency of G418-resistant colonies (weak signallers).

## 3. Results

### (a) Change in allele frequencies and evolution of sexual signal strength

When introduced into a population of weak signalling alleles at an initial frequency of 1 per cent, the strong signalling allele rapidly increased in frequency under high sexual selection but not under low sexual selection (figure 3). After 13 experimental cycles under high sexual selection, the strong signalling allele reached a mean±s.d. frequency of 85±30% and approached fixation in five out of six replicates. In the remaining replicate (high E), the strong signalling allele rose to a frequency of 69 per cent by cycle 9, before falling to 23 per cent over the next four cycles. Under low sexual selection, the frequency of the strong signalling allele remained close to the initial frequency throughout the experiment in five out of six replicates. In the remaining replicate (low D), the strong signalling allele rose to 21 per cent by the 13th cycle. Overall, the frequency of the strong signalling allele increased slightly to a mean±s.d. of 5.60±8.03% under low sexual selection.

To confirm that the large change in allele frequency caused by sexual selection corresponded to a change in the sexual signal strength, we visualized the amount of pheromone being produced by the samples of *MAT*α cells from the replicate populations under strong sexual selection at different time points in the experiment (figure 4). The amount of α-pheromone produced by signallers from each of the six replicate populations evolving under high sexual selection increased across successive experimental cycles (*t*0 to *t*13), with the exception of replicate high E. Although α-pheromone production initially increased rapidly in replicate E, it quickly decreased between experimental cycles 7 and 10.

### (b) Fitness parameters of weak and strong signallers

Three different selective forces operate in our experimental system. Sexual selection during the mating stage will favour more attractive genotypes (step 2, figure 1). Fecundity selection should favour genotypes with high mating and sporulation efficiency (steps 2–4, figure 1). Viability selection should favour haploid genotypes that exhibit high growth rates (step 5, figure 1). In the current experiment, we were only interested in sexual selection and therefore tested for differences in fecundity and viability that might have affected the results. We detected no significant difference in fecundity (*t*_8_=−0.639, *p*=0.54), as measured by the number of haploid spores generated by the sporulation of diploids arising from mating with *MATa* cells, between weak (mean±s.d.=1.328×10^6^±0.36 ×10^6^ tetrads ml^−1^) and strong signallers (mean±s.d=1.208×10^6^±0.22×10^6^ tetrads ml^−1^). That is, weak and strong signallers mated and sporulated with equal efficiency. Ancestral weak and strong signallers also did not differ in their vegetative haploid fitness (i.e. viability) when they competed under conditions that were similar to the experimental conditions, but without mating (relative fitness±s.d. of weak signaller=0.99±0.049; *t*_9_=−0.609, *p*=0.56).

As weak and strong signallers did not vary in fitness parameters subject to fecundity selection or viability selection, any change in their relative frequencies is attributable to sexual selection. Ancestral strong signallers produced more pheromone than ancestral weak signallers (figure 2) and are preferred as mates. Ancestral receivers preferred strong to weak signallers in 91.26±3.65% of matings. Preference did not change significantly over the course of the experiment; evolved receivers chose strong signallers in 89.66±3.66% of matings (*t*_34_=1.313, *p*=0.20).

## 4. Discussion

We have developed a powerful experimental system for testing genetic models of sexual selection using the yeast *S. cerevisiae*. We have used this system to demonstrate, for the first time, the central process underlying sexual selection theory: the spread of a strong signalling allele through a population of weak signallers. We found that an allele encoding a strong sexual signal rapidly increased in frequency under high sexual selection but not under low sexual selection. As we were able to demonstrate the absence of significant viability or fecundity selection on variation at the signalling locus, the increase in frequency of the strong signalling allele is attributable solely to mate choice.

It is noteworthy that evolution did not proceed identically in all six replicates of each treatment. Under high sexual selection, the strong signalling allele initially increased in frequency in all replicates, but in just one replicate it then fell rapidly. Under low sexual selection, the frequency of the strong signalling allele remained close to the initial frequency throughout the experiment, except in one replicate population where it rose slowly. This replicate-to-replicate variation is caused by the stochastic factors that can affect evolution. The rapid decline in frequency of the strong signalling allele observed in replicate high E was probably caused by selection of a novel beneficial mutation, probably linked to the weak signalling allele. If the fitness effects of this mutation were large enough to overwhelm the sexually selected advantage associated with the strong signalling allele, it would drive the spread of the weak signalling allele through the population, producing the observed dynamics. We found no difference in vegetative haploid fitness between a sample of weak and strong signallers from high E, suggesting that the mutation affected some other component of fitness (data not shown). In the laboratory, just as in nature, evolution does not necessarily follow a predictable or repeatable trajectory.

So great are the difficulties associated with the experimental evolution of sexual displays, that we are aware of only one previous study to attempt it. In an ambitious experiment, Snook *et al.* (2005) followed changes in the components of the courtship song of male *Drosophila pseudoobscura* at different intensities of sexual selection. One song component known to influence male mating success, interpulse interval, showed significant changes from baseline in both the high and low sexual selection treatments. However, interpretation of these results is hampered by an incomplete understanding of the genetic basis of courtship song and preference for it, a paucity of genetic tools and the difficulties in controlling other evolutionary forces at work during the experiment.

In this experiment, we have tested the simplest possible sexual selection scenario. We introduced variation in the strength of the sexual signal (in the form of just two alleles), but not in mate preference. Our measurements show that mate preference did not change during the course of the experiment. This could be simply because no spontaneous mutations affecting the strength of mate preference arose. Alternatively, any such mutations were not selected because they conferred too small an advantage for us to detect, or because any advantage was countered by the high cost of choice (see Pagel 1993).

Why do yeasts prefer to mate with stronger signallers? The preference for stronger pheromone sources might simply be a mechanism of locating the nearest mating partner, a system termed passive attraction (Pagel 1993). An alternative is that stronger signallers are preferred because they provide some benefit, direct or indirect, to individuals that choose them. If the pheromone is costly to produce, it may act as a reliable indicator of the producer's genetic or phenotypic quality or condition, because high-quality individuals can afford a stronger signal (Pagel 1993). The strong and weak signallers had equal fitness in our experimental conditions, so there was no detectable cost of stronger signalling, but natural conditions may be completely different. Work to test the two explanations for the existence of the preference for stronger signallers is ongoing (C. Smith, D. W. Rogers, S. J. Tazzyman, A. Pomiankowski & D. Greig 2008, unpublished data), but for the purposes of the experiment presented here, it does not matter why the preference exists, only that it does.

Our system can be used to test specific models of sexual selection. Introducing variance in mate choice in our experimental system should be relatively straightforward. The α-pheromone receptor, *STE2*, is extremely well characterized, and mutations that alter specific amino acids can change its sensitivity and specificity to α-pheromone (Sen *et al.* 1997). By adding a mutant *STE2* allele that confers either stronger or weaker preference for strong signallers to the design used herein, it would be possible to test simple two-locus two-allele models of the Fisher process (e.g. Kirkpatrick 1982). The cost of this preference could be manipulated by tightly linking the stronger preference allele to a marker conferring reduced viability. Any number of simple variations are possible, allowing the empirical testing of most existing models of sexual selection in haploids.

The study of sexual selection using traditional model organisms has perhaps been most limited by the extraordinary difficulty of measuring fitness. This is particularly true when addressing the issue of the costs of sexual signals and preferences for them. Consequently, although being able to measure and manipulate costs lies at the heart of discriminating between models of sexual selection and testing the conditions under which they work, there is very little direct empirical evidence that sexual signals and preferences impose actual costs on fitness (Kotiaho 2001). Measurement of total fitness in microbes by direct competition between genotypes is simple and powerful (Lenski *et al.* 1991). Because yeast can reproduce both sexually and asexually, sexual and non-sexual elements of fitness can be isolated. Yeast asexual fitness is very similar to Maynard Smith's (1987) concept of viability or ‘all components of fitness other than mating success’, which has subsequently been termed ‘condition’. The ability to accurately measure condition provides a powerful mechanism for testing genetic models related to the handicap principle and the exciting possibility of an experimental resolution to the lek paradox (Pomiankowski & Møller 1995; Rowe & Houle 1996).

The most compelling advantages of the use of microbes for experimental evolution come from their short generation time and large population sizes, which allows selection to act on spontaneous mutations, rather than simply sorting pre-existing alleles (e.g. Lenski & Travisano 1994). Even in the relatively short-duration experiment described here, spontaneous mutation appeared to cause replicate-to-replicate variation, and any mutations affecting signal or preference strength will be subject to selection in our experimental system. Changes in attractiveness and mate choice can be simply monitored using tester strains and the underlying genetic basis for sexual selection can be elucidated using the powerful genomic methods available for yeast (Segrè *et al.* 2006). Observing the real-time evolution of novel sexually selected traits, and preferences for them, is the ultimate test for sexual selection theory.

## Figures and Tables

**Figure 1:**
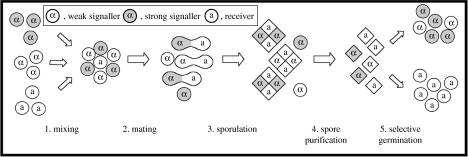
A five-step experimental cycle based on haploid models of sexual selection (cf. Barton & Turelli 1991). One mating type, *MAT*α, was designated the signaller, and the other, *MATa*, the receiver. At the start of each cycle, strong and weak signallers were mixed with receivers at a predetermined ratio and allowed to mate. The exact ratio of signallers to receivers determines the level of sexual selection. Diploids arising from these matings were forced to sporulate, producing tetrads of new haploid spores (diamonds). Any remaining unsporulated diploids and unmated haploids (circles) were destroyed by heating and the spores were allowed to germinate and grow asexually on selective media, producing separate pools of each mating type. Mating types were then mixed to start the next cycle (see §2*c* for details). This design introduces three measures to lift the restrictions on sexual selection imposed by the natural yeast mating system. First, we prevented self-fertilization by selectively germinating *MAT*α or *MATa* spores. Second, we blocked autodiploidization using heterothallic mutants incapable of switching mating types, allowing cultures of pure *MAT*α and *MATa* gametes to be propagated. Third, by manipulating the ratio of signallers to receivers away from the natural 1 : 1 ratio, we altered the amount of competition for mates, resulting in higher (or lower) levels of sexual selection.

**Figure 2:**
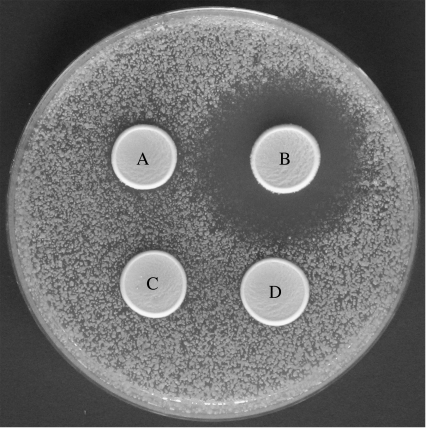
Variation in sexual signal strength in ancestral strains. α-Pheromone production was measured using the signal size assay (see §2*e*). Weak signallers (A, YDG1093) produced a much smaller halo than did strong signallers (B, YDG1074). The signalling locus is silent in *MATa* cells and therefore no halo was observed around receivers carrying the weak signalling allele (C, YDG1096) or the strong signalling allele (D, YDG1075).

**Figure 3:**
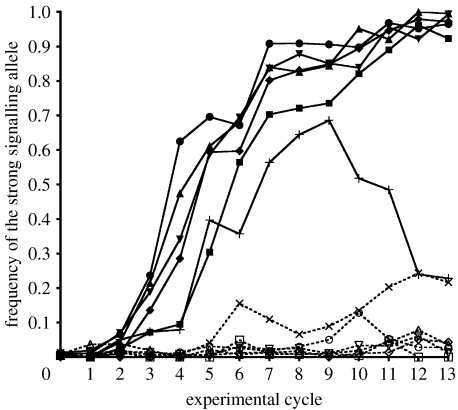
Evolution at the signalling locus under different levels of sexual selection. Change in frequency of the initially rare (1%) strong signalling allele in six replicate populations exposed to high sexual selection (solid lines) and six replicate populations exposed to low sexual selection (dotted lines). Each replicate of each treatment is indicated by a different symbol. High sexual selection: filled up triangles, A; filled down triangles, B; filled diamonds, C; filled circles, D; pluses E; filled squares, F. Low sexual selection: open squares, A; open diamonds, B; down triangles, C; crosses, D; open circles, E; up triangles, F.

**Figure 4:**
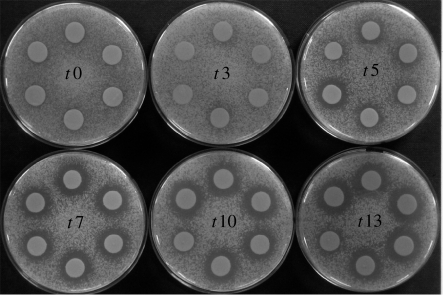
Evolution of sexual signal strength in populations under high sexual selection treatment. Signal strength (α-pheromone production) is shown by the size of the dark ‘halo’ surrounding each round patch of signalling cells. The halo is caused by the inhibition of growth of an overlaid lawn of *MATa* tester cells that are hypersensitive to α-pheromone and cannot grow when it is at a high concentration. Thus, a larger halo indicates greater α-pheromone production. Replicates are ordered clockwise from A to F starting at the top of each plate. Results are shown only for the initial population (*t*0) and experimental cycles 3, 5, 7, 10 and 13.
